# Continuous Infusion of Iohexol to Monitor Perioperative Glomerular Filtration Rate

**DOI:** 10.1155/2022/8267829

**Published:** 2022-05-24

**Authors:** Kjellbjørn Jakobsen, Bjørn O. Eriksen, Ole M. Fuskevåg, Stephen J. Hodges, Lars M. Ytrebø

**Affiliations:** ^1^Anesthesia and Critical Care Research Group, UiT – The Arctic University of Norway, Tromsø, Norway; ^2^Metabolic and Renal Research Group, UiT - the Arctic University of Norway, Tromsø, Norway; ^3^Section of Nephrology, Clinic of Internal Medicine, University Hospital of North Norway, Tromsø, Norway; ^4^Department of Laboratory Medicine, Division of Diagnostic Services, University Hospital of North Norway, Tromsø, Norway; ^5^Division of Surgical Medicine and Intensive Care, University Hospital of North Norway, Tromsø, Norway

## Abstract

Continuous monitoring of the glomerular filtration rate (GFR) in the perioperative setting could provide valuable information about acute kidney injury risk for both clinical and research purposes. This pilot study aimed to demonstrate that GFR measurement by a continuous 72 hrs iohexol infusion in patients undergoing colorectal cancer surgery is feasible. Four patients undergoing robot-assisted colorectal cancer surgery were recruited from elective surgery listings. GFR was determined preoperatively by the single-sample iohexol clearance method, and postoperatively at timed intervals by a continuous iohexol infusion for 72 hrs. Plasma concentrations of creatinine and cystatin C were measured concurrently. GFR was calculated as (iohexol infusion rate (mg/min))/(plasma iohexol concentration (mg/mL)). The association of the three different filtration markers and GFR with time were analysed in generalized additive mixed models. The continuous infusion of iohexol was established in all four patients and maintained throughout the study period without interfering with ordinary postoperative care. Postoperative GFR at 2 hours were elevated compared to the preoperative measurements for patients 1, 2, and 3, but not for patient 4. Whereas patients 1, 2, and 3 had u-shaped postoperative mGFR curves, patient 4 demonstrated a linear increase in mGFR with time. We conclude that obtaining continuous measurements of GFR in the postoperative setting is feasible and can detect variations in GFR. The method can be used as a tool to track perioperative changes in renal function.

## 1. Introduction

The considerable morbidity and mortality associated with perioperative acute kidney injury (AKI) is well documented, but little progress has been made in the prevention and treatment of this condition [1, 2]. One reason may be insufficient understanding of renal pathophysiology due to lack of tools for continuous monitoring of overall kidney function in this setting. Whereas measured glomerular filtration rate (mGFR) is probably the best overall indicator of renal function, cystatin C and creatinine are more often used for monitoring GFR after surgery. Although serum creatinine is included in the current AKI definition [3], both these markers are known to be unreliable in the perioperative setting [1, 4]. Creatinine does not rise before GFR is significantly reduced [5], which causes delay in the diagnostic process. Accurate determination of GFR is also important for drug dosing, particularly for drugs with a narrow therapeutic window.

The plasma clearance of iohexol has been established as reliable tool for measuring GFR in the steady state condition [5]. Recently, Dixon et al. found that a continuous low-dose iohexol infusion could detect changes in GFR in intensive care patients at high risk for AKI [6]. In the present study, we aimed to demonstrate that the method can also monitor GFR variations in patients without AKI risk-factors undergoing robot-assisted colorectal cancer surgery during the first 72 hrs of the postoperative period.

## 2. Methods

Four patients scheduled for elective robot-assisted colorectal cancer surgery were recruited. Informed, signed consent was obtained from the patients before inclusion in the study. This study (2018/19347/REK) was approved by the regional ethical committee and was registered at http://www.clinicaltrials.gov (clinicalTrials.gov ID NCT03881332).

### 2.1. Inclusion Criteria

Patients ≥60 years old scheduled for elective robot-assisted laparoscopic colorectal cancer surgery at the University Hospital of North Norway, Tromsø. All four patients were treated in accordance to the Enhanced Recovery After Surgery (ERAS) guidelines [7].

### 2.2. Exclusion Criteria

Exclusion criteria are as follows: inability to provide informed consent prior to elective surgery; complicating secondary metabolic diseases, such as diabetes; a radiological examination using contrast within a week before surgery; allergy to radiocontrast media; GFR <45 mL/min; patients taking drugs which could potentially interact with iohexol (metformin if SCr >150 *μ*mol/L, phenothiazines, monoamine oxidase inhibitors, levo-thyroxine, amiodarone, interleukin-2 agents, and Tc99m-MDP); disorders in which iohexol may potentially interfere with monitoring (thyroid disease, myasthenia gravis, and phaeochromocytoma); hyperviscosity disorders (sickle cell disease, homocystinuria, and multiple myeloma); pregnancy or breast-feeding.

### 2.3. Preoperative Single Injection Iohexol Administration and Sampling

Baseline GFR was determined one day before surgery by single-sample plasma clearance of iohexol as previously described [8, 9]. The patients were instructed to avoid large meals with meat and nonsteroid anti-inflammatory drugs two days before the investigation, and performed after overnight fasting, including abstinence from nicotine-containing products. The subjects were reminded to not restrict their intake of water. On the morning of measurement, a 3-lumen central venous catheter (CVC) was inserted and baseline venous blood samples were collected. A total of 5 mL of iohexol (Omnipaque, 300 mg/mL; Amersham Health, London, UK) was then subsequently injected intravenously and the catheter flushed with 20 mL isotonic saline. The subjects were monitored for allergic reactions for 30 min and then allowed to walk about freely and eat a light breakfast, but meat and smoking were restricted. To ensure complete distribution of iohexol in the extracellular fluid volume, the shortest sampling time was set at 180 min. The exact time from injection to sampling was measured in minutes using a stopwatch. Iohexol (Omnipaque 300®) from one batch purchased from Amersham was used.

### 2.4. Continuous Iohexol Administration and Sampling

Iohexol was administered via a dedicated line of the CVC catheter. The giving set and syringe containing iohexol was covered in a light-impermeable sheath. After surgery and within 1 hour after the patient had been admitted to the postanaesthesia care facility, a loading dose of 2 mL iohexol was administrated intravenously. This was followed by a continuous infusion of iohexol at 0.5 mL/h (343.5 mg/h) for 72 h via a syringe pump, as described by Dixon et al. [6]. Volumetric mean accuracy in these pumps were ±2% according to the manufacturer. Plasma samples were obtained for plasma clearance measurements (Cl_P_) at 30 mins, 1 h, 2 h, and 4 h at the day of surgery, and at 08 : 00 h, 10 : 00 h, 18 : 00 h, and 20 : 00 h on subsequent days for up to 72 hrs. Sampling was performed from a separate line of the CVC catheter after the syringe pump had been stopped for 1 minute. Time zero was the time of commencing the continuous infusion of iohexol. Cl_P_ was calculated by the formula: Cl_P_ (mL/min) = (iohexol infusion rate (mg/min))/(plasma iohexol concentration (mg/mL)). Processing and analyses of samples were performed at the local hospital laboratory. Plasma iohexol was measured with liquid chromatography with tandem mass spectrometry. A detailed description of the methodologies for the laboratory procedures are provided in the Supplementary Methods.

### 2.5. Creatinine and Cystatin C Sampling

Samples for analysis of creatinine and cystatin C were collected at the same time as plasma samples for plasma clearance measurements of iohexol.

### 2.6. Statistics

Relative changes in concentration of iohexol, creatinine, and cystatin C were calculated as (actual measure – 2 hrs postoperative measure)/2 hrs postoperative measure.

The dependence of the three different filtration markers and mGFR with time was analysed in generalized additive mixed models (GAMMs), which allows for nonlinear relationships and included a random intercept for each patient [10]. The Akaike Information Criterion (AIC) was used to compare the fit of different models. The analyses were performed with the mgcv-package in R version 4.0.5 (https://www.r-project.org). Statistical significance was set at *p* < 0.05.

## 3. Results

Demographic data, including baseline single-sample iohexol clearance, are shown in Table 1. All four patients were treated in accordance to the study protocol. Between-day coefficient of variation (CV) for the iohexol assay was 5.4% on four consecutive days. Intraday CV was 2.8%. The infusion of iohexol was never discontinued neither did a change in the infusion rate of iohexol occur during the study period.Vascular access for sampling was achieved in all four patients using an indwelling central venous catheter (CVC). Ordinary postoperative care was performed as usual without interference from the GFR measurements.

**Figure 1:**
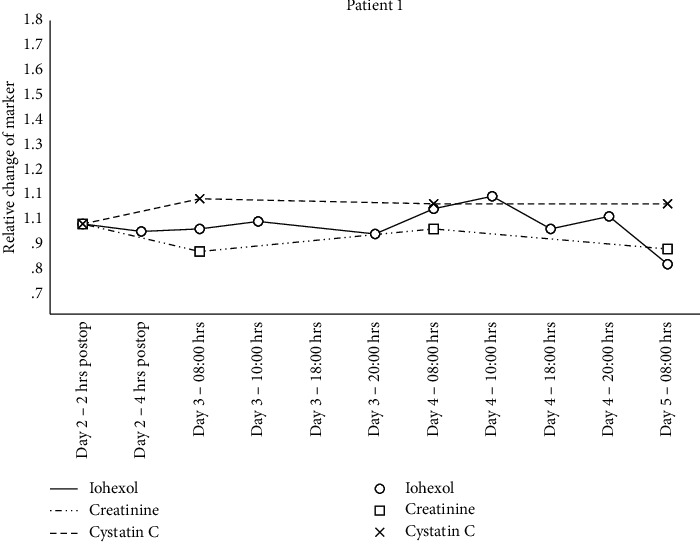
Relative change in plasma concentration of iohexol, creatinine, and cystatin C for patient 1.

**Figure 2:**
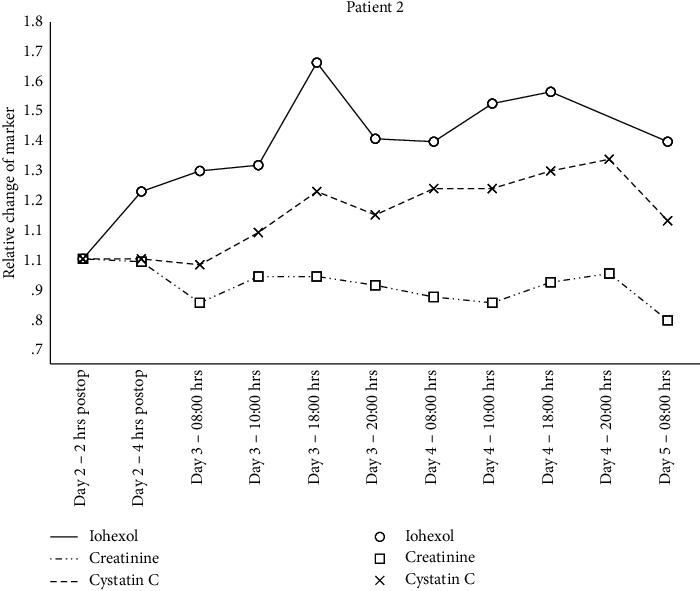
Relative change in plasma concentration of iohexol, creatinine, and cystatin C for patient 2.

**Figure 3:**
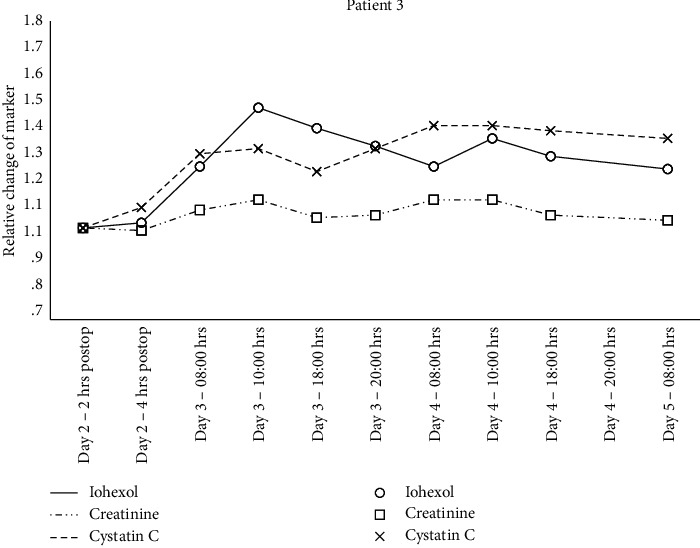
Relative change in plasma concentration of iohexol, creatinine, and cystatin C for patient 3.

**Figure 4:**
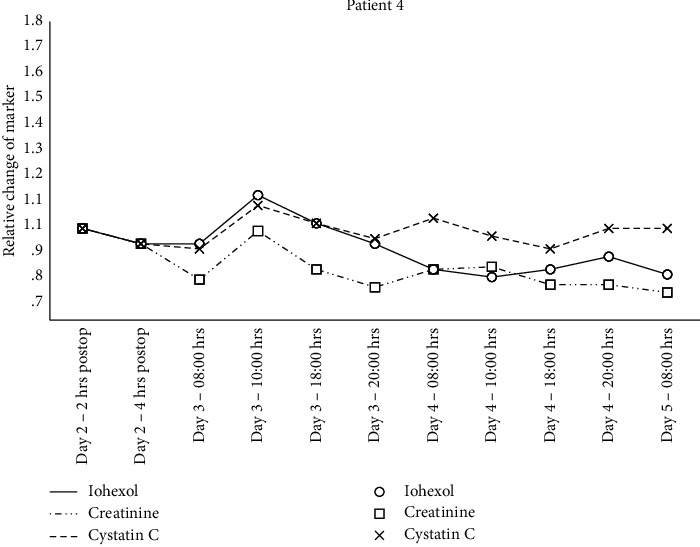
Relative change in plasma concentration of iohexol, creatinine, and cystatin C for patient 4.

**Figure 5:**
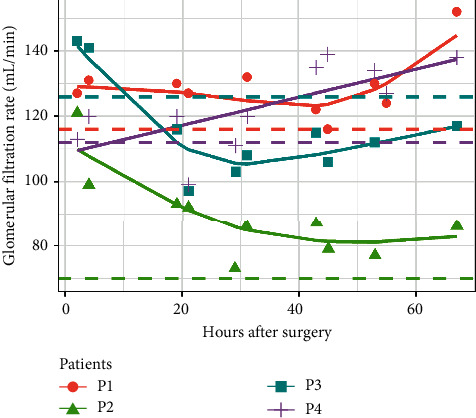
Time dependency of measured glomerular filtration rate (mGFR) calculated from the iohexol measurements analysed by generalized additive mixed models (GAMMs). The markers indicate individual measurements of GFR. The solid lines indicate fitted values for each patient. Preoperative mGFRs are indicated by dashed horizontal lines.

Individual changes in creatinine, cystatin C, and iohexol relative to the 2 hrs. postsurgery sample are shown in Figures 1–4 and mGFR data are shown in Figure 5.

A GAMM with separate relationships for each patient demonstrated statistically significant associations between the plasma iohexol concentration and time for patient 2 (*p* < 0.001), 3 (*p*=0.008), and 4 (*p*=0.01) but not for patient 1 (*p*=0.78). The same model with creatinine as the dependent variable found statistically significant associations for patient 2 (*p*=0.005) and 4 (*p* < 0.001) but not for patient 1 (*p*=0.97) and 3 (*p*=0.39). Cystatin C as the dependent variable resulted in statistically significant associations for patient 2 (*p* < 0.001) and 3 (*p* < 0.001) but not for patient 1 (*p*=0.63) and 4 (*p*=0.93).

The time dependency of mGFR was analysed in the same GAMM. A model with separate nonlinear relationships for each of the four patients had a better fit to the data than a common nonlinear relationship fitted to all the patients (AIC 333.7 vs. 341.5). The fitted values for the best model were plotted (Figure 5), where the preoperative mGFR are also indicated by dashed horizontal lines. The first postoperative mGFR at 2 hours were considerably elevated compared to the preoperative measurements for patients 1, 2, and 3 but not for patient 4. Whereas patients 1, 2, and 3 had u-shaped postoperative GFR curves, patient 4 demonstrated a linear increase in GFR with time. In comparison, serum creatinine did not markedly reflect these dynamic renal changes.

## 4. Discussion

The present pilot study demonstrated the feasibility of continuous measurements of GFR by continuous 72 hrs infusion of iohexol in patients undergoing robot-assisted colorectal cancer surgery. Following the study by Dixon et al., this investigation confirms that GFR measurements can be obtained in dynamic clinical situations where GFR varies over short time periods. We extend the results of Dixon et al. by showing that the method can also detect smaller variations in GFR in patients without high risk of AKI. The GFR measurements did not delay other clinical interventions or investigations.

Blood samples were immediately brought to the laboratory, spun, and kept at −70°C until further processing. The time interval between when the blood samples were obtained and frozen where <20 minutes. Sampling, preanalysis preparation, analysis, and postanalysis of iohexol took approximately 2 hours in this study. However, as a clinical routine service, mGFR can be provided within 30 minutes after the sample arrives to the laboratory, making a swift response to alterations in renal function possible.

The use of iohexol for measuring GFR is safe. The fear of nephrotoxicity using iohexol stems from the risk of contrast-induced nephropathy when the substance is used as a radiocontrast agent, but the doses of iohexol injected for the measurement of GFR are much smaller than that used for CT scans or for coronary interventions [11]. The small doses used for single-sample GFR have not been demonstrated to be nephrotoxic even in patients with GFR <20 mL/min/1.73 m^2^ [12]. The volume of iohexol used for the CILDI protocol is less than half that of a proposed threshold ratio that needs to be exceeded for contrast-assosciated AKI to developing a 40 kg individual [13]. None of our patients developed AKI as determined by changes in creatinine using the KDIGO criteria for AKI [2], neither were there any signs of decreased urinary output for any of the patients.

The methods detected statistically significant variations in GFR in the postoperative period consistent with the different trajectories of GFR in the four patients. GFR rose postoperatively for patients 1, 2, and 3. Dixon et al. defined a variation in GFR >10.3% as clinically relevant [13]. According to this criterion, the increase in GFR for these three patients indicates a clinically relevant change in GFR. eGFR would not alert the clinician to those dynamic changes. The pathophysiology of these variations and their implications for AKI risk will be the subject of planned research projects. In particular, the importance of postoperative renal hyperfiltration needs further exploration [14].

## 5. Conclusion

We conclude that continuous GFR monitoring over an extended time period is feasible and can be used for investigations of postoperative changes in renal function. We acknowledge that continuous infusion of iohexol is unlikely to become a clinically routine methodology, but it may become a useful tool in evaluation of renal function in patients at high risk of AKI.

## Figures and Tables

**Table 1:** Patient characteristics and perioperative data.

	Patient 1	Patient 2	Patient 3	Patient 4
Baseline single-sample GFR^*∗*^ (mL/min)	116	70	126	112
Baseline single-sample GFR^*∗*^ (mL/min/1.73 m^2^)	97	59	105	100
Baseline eGFR creatinine^†^ (mL/min/1.73 m^2^)	97	68	91	92
Baseline eGFR cystatin C^‡^ (mL/min/1.73 m^2^)	97	61	96	78
Baseline eGFR crea-cysC^§^ (mL/min/1.73 m^2^)	94	65	95	85
Sex (M/F)	F	M	M	M
Age (years)	60	81	66	63
Body mass index (kg/m^2^)	29	31	26	27
Weight preoperative (kg)	90	91	86	80
Weight gain (kg)	3.6	—	—	1.6
Height (cm)	176	172	183	173
ASA	2	3	2	2
Preexisting diabetes	No	No	No	No
Preexisting cardiovascular disease	No	Yes	No	No
Blood loss (mL)	100	150	100	80
Length of surgery (min)	270	255	350	205
Intraoperative fluids given (mL)	1300	1300	1100	1000

^*∗*^GFR: glomerular filtration rate; ^†^eGFR creatinine: estimated GFR CKD-EPI creatinine (2009); ^‡^eGFR cystatin C: estimated GFR CKD-EPI cystatin C (2012); ^§^eGFR crea-cysC: estimated GFR CKD-EPI creatinine-cystatin C (2012), ASA: American Society of Anesthesiologists classification.

## Data Availability

The data supporting the conclusions of the study can be obtained upon reasonable request by reaching out to the corresponding author.

## Supplementary Materials

Supplementary Materials Chemicals and solutions. Determination of iohexol in human serum. Precision and accuracy. Determination of creatinine and cystatin C in human serum.
