# Supplementary material for: Continuous Infusion of Iohexol to Monitor Perioperative Glomerular Filtration Rate
Source: Int J Nephrol. 2022 May 24;2022:8267829. doi: 10.1155/2022/8267829 (PMC9155923; doi:10.1155/2022/8267829)
Supplement: Supplementary Materials — Chemicals and solutions. Determination of iohexol in human serum. Precision and accuracy. Determination of creatinine and cystatin C in human serum. [file 8267829.f1.docx]

Supplementary Methods - Laboratory Procedures

*Chemicals and solutions*

Iohexol and Iohexol-d5 was obtained from Toronto Research Chemicals Inc. (Ontario, Canada) and iohexol for quality controls (QCs) was purchased from TCI Chemicals (Tokyo, Japan). LC-MS grade methanol was purchased from Honeywell™ Riedel-de Häen™ (Seelze, Germany). LC-MS grade formic acid was obtained from Fluka (Sigma-Aldrich, St. Louis, MO). Ultrapure water (18.2 MΩ) from Millipore Advantage Milli-Q system (Millipore SAS, Molsheim, France) was used for all the analyses.

*Determination of iohexol in human serum*

Two stock solutions of iohexol were prepared in methanol and stored at -30 °C. A 6-point calibration curve and two QCs for iohexol was constructed in drug-free serum (1-240 mg/l). A Tecan Freedom Evo 200 (Männedorf, Switzerland) liquid handling workstation was used for sample preparation. Calibrators, QCs and samples (50 µL) were prepared by adding 50 μl internal standard (aqueous iohexol-d5, 3.3mg/L) in a 96-well MegaBlock® 1.2 mL, PP, (Sarstedt, Germany). To each of the wells 0.5 mL ice-cold methanol was added. The plate was mixed on a Bioshake (Quantifoil Instruments, Jena, Germany) at 1500 rpm for 3 min and centrifuged at 240 x g for 8 min (Hettich Rotina 320R, Tuttlingen, Germany). 100 µl of the supernatant was transferred to a 96-well collection plate (Waters, Milford, MA). After sealing of the plate, 0.1 µl of the supernatant was injected to the LC-MS/MS system and analyzed by LC-MS/MS using a Waters Acquity UPLC I-Class FTN system with an autosampler and a binary solvent delivery system (Waters, Milford, MA) interfaced to Waters Xevo TQ-S benchtop tandem quadrupole mass spectrometer (Waters, Manchester, UK). The chromatography was performed on a 2.1 x 100 mm Waters Acquity Cortecs® T3, 1.6 µm column. Eluent A consisted of 0.1% formic acid in water; eluent B consisted of 0.1% formic acid in methanol. Gradient elution was performed with 2% B at start and had a linear increase to 60% B until 0.6 min, a linear increase to 98% B until 1.5 min, and re-equilibration until 2.7 min with 1% B. The flow rate was 0.3 mL/min and the column temperature was maintained at 50 °C. The mass spectrometer was operated in positive electrospray ion mode and spray voltage was set to 0.9 kV. The system was controlled by MassLynx version 4.1 software. Desolvation gas temperature was 500 °C; source temperature was 150 °C; desolvation gas flow was 1000 L/h; cone gas flow was 150 L/h; collision gas pressure was 4 x 10-3 mBar. For quantitative analysis of iohexol the following multiple reaction monitoring (MRM) transitions were used (bold transitions are qualifiers): m/z 821.9->803.8/**602.4** and 826.9->808.8/**607.5** (iohexol and iohexol-d5).

*Precision and accuracy*

The method was validated and was found to be linear from 1.5 to at least 240 mg/L (r2 > 0.999). Lower limit of quantification was found to be 0.5 mg/L (0.1 µl injection volume). Between-day coefficient of variation (CV) for iohexol was 5.4% on four consecutive days. CV for intraday precision value was 2.8 % and was calculated by assaying three samples (low, medium and high concentration) six times on the same day. Accuracy for recovery test was 91.1-107.9 % (9 levels, n = 3 for each). Additionally, the quality is assured through the Equalis external quality assessment program for iohexol four times a year. Single-sample iohexol clearance was calculated as described by Eriksen et al. (S1).

*Determination of creatinine and cystatin C in human serum*

Serum creatinine was measured using an enzymatic assay standardized to the isotope dilution mass spectrometry method (CREA Plus, Roche Diagnostics). Cystatin C was analyzed with a particle enhanced turbidimetric immunoassay with reagents from Gentian (Gentian, Moss, Norway) and a Modular E analyzer (Roche Diagnostics). The cystatin C measurements were then recalibrated to the international reference standard using a Cobas 8000 (Roche Diagnostics). The 2009 CKD-EPI Creatinine and the 2012 CKD-EPI Cystatin C and CKD-EPI Creatinine-Cystatin C equations were applied to estimate GFR (S2, S3).

**Supplementary References**

S1. Eriksen BO, Melsom T, Mathisen UD, et al. GFR normalized to total body water allows comparisons across genders and body sizes. J Am Soc Nephrol. 2011;22(8):1517-25.

S2. Levey AS, Stevens LA, Schmid CH, et al. A new equation to estimate glomerular filtration rate. Ann Intern Med. 2009;150(9):604-12.

S3. Inker LA, Schmid CH, Tighiouart H, et al. Estimating glomerular filtration rate from serum creatinine and cystatin C. N Engl J Med. 2012;367(1):20-9.
